# Design, Synthesis, Molecular Dynamics, and In Vitro Evaluation of Isatin‐Based Quinazoline Derivatives as Potential Antidiabetic Agents

**DOI:** 10.1002/cbdv.71694

**Published:** 2026-09-10

**Authors:** Sahil Jaidka, Ankush Kumar, Christopher Basumatary, Thakur Gurjeet Singh, Rohit Bhatia

**Affiliations:** ^1^ Chitkara College of Pharmacy Chitkara University Rajpura Punjab India; ^2^ Department of Pharmaceutical Engineering and Technology IIT BHU Varanasi India

**Keywords:** antidiabetic agents, indoloquinazolinedione, MD simulation, molecular docking, α‐amylase inhibition

## Abstract

A series of substituted indolo[2,1‐b]quinazoline‐6,12‐diones (SJ1‐SJ8), which consist of already published derivatives (SJ1‐SJ5) and newly synthesized derivatives (SJ6‐SJ8), has been studied for their possible α‐amylase inhibition property as an antidiabetic lead compound. The synthesized derivatives have been identified by spectroscopy and analysis methods and tested for their in vitro α‐amylase inhibitory activity. The most potent inhibitory activity among the tested derivatives was showed by SJ2 (IC_50_ = 18.49 ± 0.06 µM), similar to the reference drug acarbose (IC_50_ = 16.26 ± 0.02 µM). The second‐best activity was observed for the newly identified derivative SJ8 (IC_50_ = 19.67 ± 0.19 µM). Molecular docking and 100 ns molecular dynamics simulations supported favorable and stable interactions of SJ2 in the α‐amylase active site, while MM‐PBSA analysis showed favorable binding energetics. The electronic properties of SJ2 were also clarified by DFT and molecular electrostatic potential analyzes, and in silico ADMET profiling indicated promising predicted drug‐like and pharmacokinetic properties. To summarize, SJ2 is a promising lead for α‐amylase inhibition, which needs further optimization and biological validation. Future evaluation against α‐glucosidase and relevant in vivo models will be essential to determine the more general antidiabetic potential of this scaffold.

## Graphical abstract

A series of substituted indolo[2,1‐b]quinazoline‐6,12‐diones (SJ1‐SJ8) has been studied for their possible α‐amylase inhibition property as an antidiabetic lead compound. The most potent inhibitory activity among the tested derivatives was showed by SJ2 (IC_50_ = 18.49 ± 0.06 µM), similar to the reference drug acarbose (IC_50_ = 16.26 ± 0.02 µM).

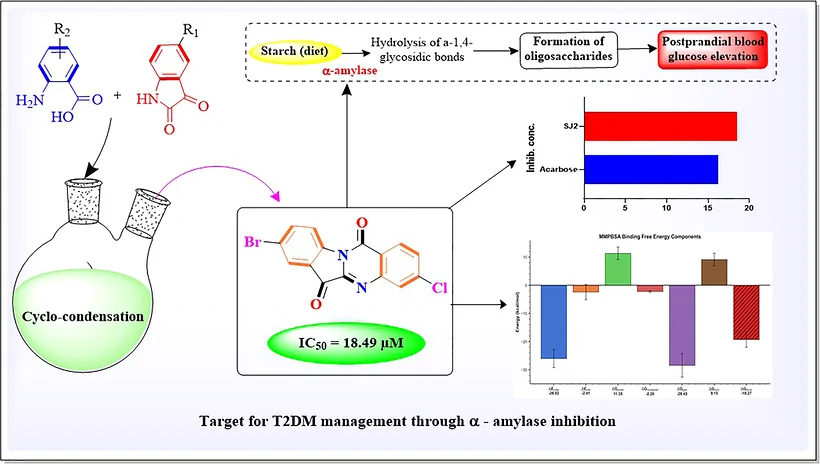

**Abbreviations**
AcOH: acetic acid
Ar: aromatic ring
CADD: computer‐aided drug design
cm^−^^1^
: reciprocal centimeter
DCM: dichloromethane
DMF: dimethylformamide
DMSO: dimethyl sulfoxide
eq.: equivalent
EtOAc: ethyl acetate
FTIR: Fourier transform infrared spectroscopy
HRMS: high‐resolution mass spectrometry
Hz: hertz
IC_50_
: half maximal inhibitory concentration
IR: infrared
J: coupling constant
MD: molecular dynamics
MeOH: methanol
MHz: megahertz
NMR: nuclear magnetic resonance
PCA: principal component analysis
PPA: polyphosphoric acid
ppm: parts per million
Rf: retention factor
RMSD: root mean square deviation
RMSF: root mean square fluctuation
rpm: revolutions per minute
SASA: solvent accessible surface area
SOCl_2_
: thionyl chloride
TLC: thin layer chromatography
UV: ultraviolet spectroscopy

## 1 Introduction

**FIGURE 1:**
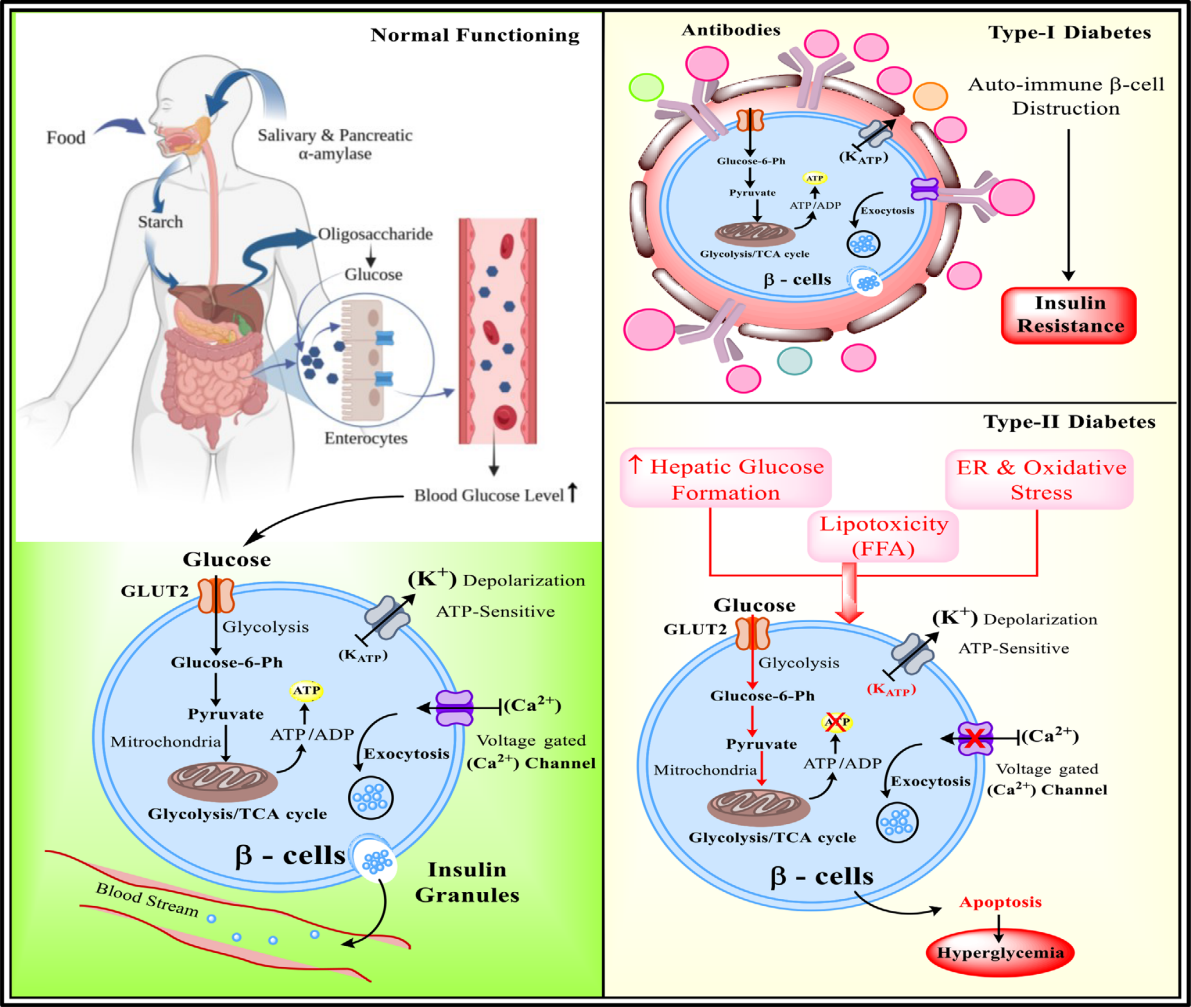
Understanding the mechanisms: normal physiology versus Type‐I and Type‐II diabetes.

Type 2 Diabetes Mellitus (T2DM) is a global metabolic emergency [1], with the International Diabetes Federation (IDF) predicting 853 million cases by 2050 [2], putting an unsustainable strain on healthcare infrastructure globally [3, 4], particularly in low‐ and middle‐income nations [5]. T2DM is characterized by progressive β‐cell dysfunction, impaired insulin secretion, peripheral insulin resistance, and chronic hyperglycemia (as displayed in Figure 1) [6, 7, 8]. This leads to a variety of macrovascular and microvascular complications, including cardiovascular disease, nephropathy, retinopathy, and neuropathy [9]. Metformin, sulfonylureas, DPP‐4 inhibitors, GLP‐1 receptor agonists, and SGLT‐2 inhibitors are among the current pharmacotherapeutic treatments [10]. Despite this wide range of alternatives, several clinical constraints remain: GI discomfort (metformin), hypoglycemic risk (sulfonylureas), low glycemic effectiveness and financial obstacles that limit availability in developing countries [11]. Furthermore, many treatments focus on insulin secretion or sensitivity pathways rather than the upstream enzymatic mechanisms that control postprandial glucose excursions [8]. Inhibiting pancreatic α‐amylase, the starch‐hydrolyzing enzyme in the small intestine lumen, is a unique and therapeutically relevant strategy for reducing postprandial hyperglycemia [12, 13]. α‐amylase inhibitors lower glycemic load without relying on insulin‐dependent pathways, reducing the risk of hypoglycemia [14]. Acarbose, the classic α‐amylase inhibitor, has glycemic effectiveness but is restricted by gastrointestinal side effects due to its non‐absorbed oligosaccharide composition [15]. This highlights the unmet need for structurally innovative, drug‐like small compounds with high tolerability and equivalent or greater inhibitory efficacy [16].

Heterocyclic scaffolds play an important role in antidiabetic drug development [17], with nitrogen‐containing fused ring structures displaying favorable interactions inside enzyme active sites via hydrogen bonding, π‐π stacking and van der Waals contacts [18, 19]. Among them, indole and quinazoline are two of the most important cores in medicinal chemistry [11, 20]. Indole analogues have shown broad‐spectrum bioactivity [21], including antidiabetic [22, 23], anticancer, anti‐inflammatory and antiviral activities, which are due to the scaffold's ability to stack planar aromatics and its bioisosteric resemblance of relevant aromatic moieties [24]. Quinazoline derivatives are approved medicines that inhibit enzymes, such as α‐amylase [25], α‐glucosidase [26], aldose reductase and DPP‐4 [27]. They use nitrogen atoms to form directed hydrogen bonds [28, 29].

**FIGURE 2:**
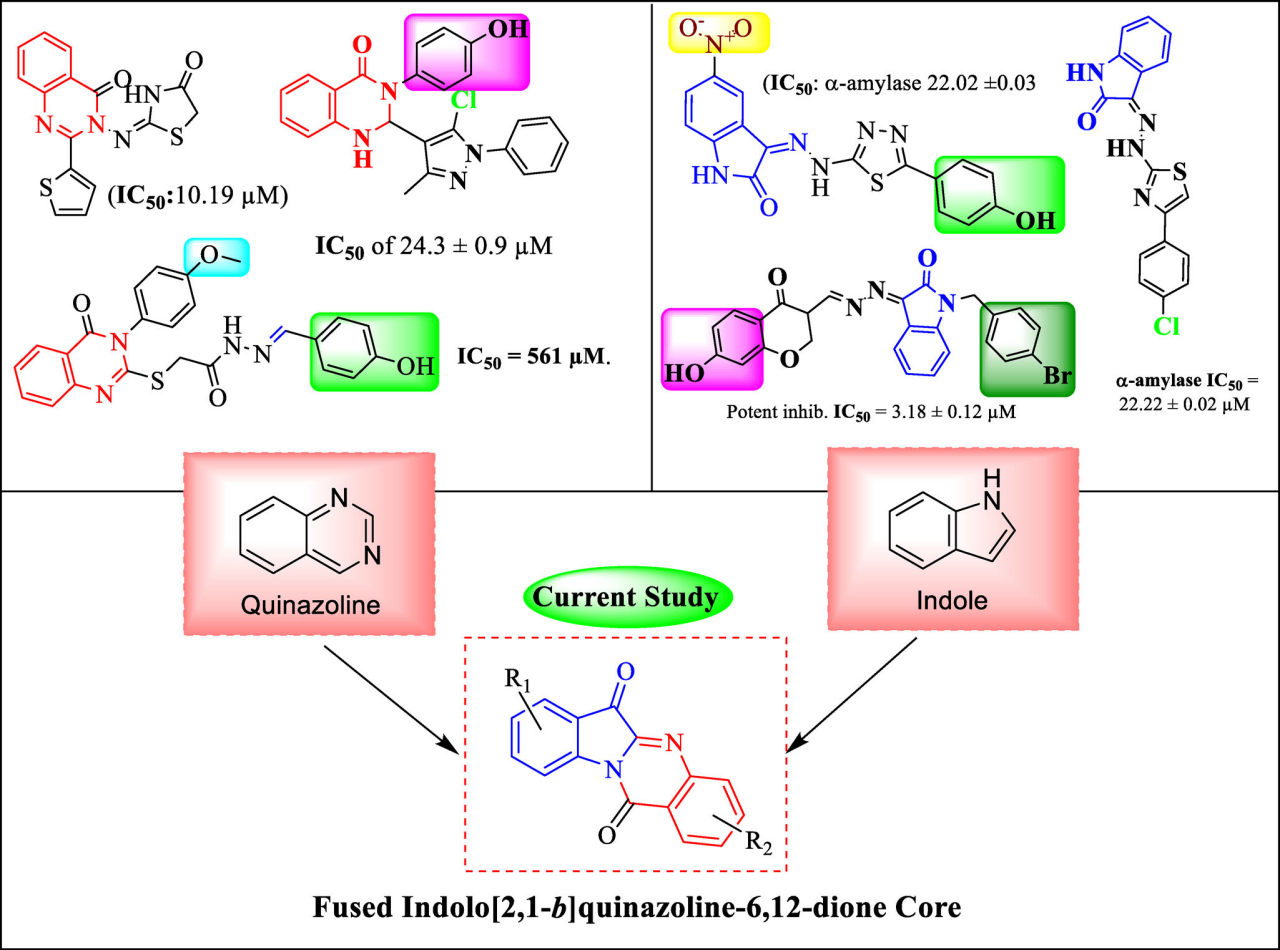
Design strategy for substituted isatin‐based quinazoline hybrids as potential α‐amylase inhibitors.

The strategic fusion of these two pharmacophores into the indolo[2,1‐*b*]quinazoline‐6,12‐dione (tryptanthrin‐type) scaffold results in a rigid, planar tetracyclic aromatic system with dual carbonyl functionalities at C‐6 and C‐12 [30, 31], as well as accessible substitution sites that allow for systematic medicinal chemistry exploration [32] (as shown in Figure 2). This design increases the π‐surface area for enzyme active‐site interaction, offers directed hydrogen bond acceptors and allows for halogen‐dependent electronic and steric manipulation [33, 34]. Tryptanthrin is a naturally occurring indoloquinazolinedione with known enzyme inhibitory action, establishing a pharmacological precedence for this class.

This study presents the design, synthesis, and comprehensive biological and computational characterization of a series of substituted indoloquinazolinedione derivatives (SJ1‐SJ8) as α‐amylase inhibitors, incorporating in vitro bioassay, molecular docking, 100 ns molecular dynamics simulation, MM‐PBSA free energy analysis, DFT electronic characterization and ADMET profiling into a mechanistically cohesive narrative to support the identification and optimization of SJ2 as a lead antidiabetic candidate.

The biological activities of tryptanthrin and related indoloquinazoline derivatives have been studied previously, but there has been little systematic evaluation of these compounds as α‐amylase inhibitors. The present work extends this scaffold to α‐amylase inhibition by investigating the effect of C‐8 and C‐2 substitution patterns. In particular, the identification of SJ2 as an active derivative provides a basis for further optimization of this scaffold as an α‐amylase inhibitory lead.

We report here the design, synthesis, and biological and computational characterization of a series of substituted indoloquinazolinedione derivatives (SJ1‐SJ8) as α‐amylase inhibitors. The series contains five previously reported derivatives (SJ1–SJ5) [35, 36, 37, 38, 39] and three newly identified derivatives (SJ6–SJ8), which allow the investigation of the influence of structural substitution on α‐amylase inhibitory activity. The study combines in vitro bioassay, molecular docking, 100 ns molecular dynamics simulation, MM‐PBSA free energy analysis, DFT electronic characterization, and ADMET profiling to provide a mechanistic framework for the identification and optimization of promising α‐amylase inhibitory leads. SJ2 is identified as the most active derivative.

## 2 Results and Discussion

### 2.1 Chemistry

**SCHEME 1:**
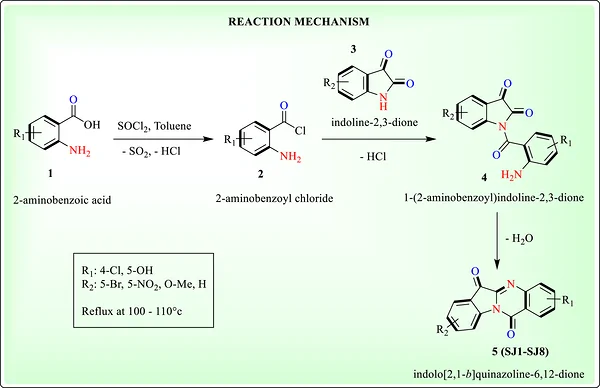
General mechanism of the purposed reaction for formation of compound (SJ1‐SJ8).

Through systematic variation of the substituent groups on the starting compounds, a general approach for synthesis has provided a number of structurally diverse indoloquinazoline analogues (SJ1‐SJ8). According to a structure‐based literature search on CAS SciFinder, SJ1‐SJ5 have been found to be already reported derivatives, while SJ6‐SJ8 could not be found in any of the existing literature and hence have been classified as new derivatives in the current study. This series of compounds has been studied to understand how the variations in electronic, steric and hydrogen bonding properties affect the biological activity of the indoloquinazoline core. To verify their chemical structures and purity, the synthesized derivatives were thoroughly characterized and purified using a variety of spectroscopic and analytical methods [40]. Characteristic functional groups, specifically the carbonyl (C═O), aromatic C═C, C‐N and N‐H stretching vibrations, were identified using Fourier‐transform infrared spectroscopy (FT‐IR). To clarify the proton environment and validate the aromatic and heterocyclic proton framework of the synthesized compounds ^1^H NMR spectroscopy was employed. The carbon skeleton was further established using ^13^C NMR spectroscopy, which further confirmed the presence of important carbonyl and quaternary carbons in the fused indoloquinazoline system. In order to establish the molecular formulas of the synthesized compounds and the effective synthesis of the intended indoloquinazoline derivatives, HRMS analysis was also performed to ascertain the precise molecular masses of the compounds (as seen in Scheme 1).

### 2.2 In Vitro Evaluation (α‐Amylase Inhibition)

The synthesized indolo[2,1‐*b*]quinazoline‐6,12‐dione derivatives (SJ1‐SJ8) were tested for their in vitro α‐amylase inhibitory activity and were expressed in terms of IC_50_ values (µM). The test was validated using the standard medication Acarbose. Lower IC_50_ values imply significant enzyme inhibition. SJ2 (18.49 ± 0.06 µM) and SJ8 (19.67 ± 0.19 µM) showed the most inhibitory efficacy among the investigated drugs and their IC_50_ values were similar to those of the standard acarbose (16.26 ± 0.02 µM). These results suggest that these compounds have considerable α‐amylase inhibitory potential and might be good lead candidates for additional optimization. In vitro α‐amylase inhibition study was the most crucial experiment to verify the biological properties of the synthesized analogs, and the remaining computational studies were carried out to understand the mechanism of the inhibitory effect, especially for SJ2. Moderate inhibitory action was shown by compounds SJ6 (24.79 ± 0.60 µM), SJ3 (31.08 ± 0.12 µM), SJ7 (32.74 ± 0.20 µM), and SJ4 (39.55 ± 0.80 µM). The difference in their IC_50_ values indicates that structural changes affect the efficacy of enzyme binding, perhaps as a result of variations in the electronic characteristics and steric orientation of substituents.

**FIGURE 3:**
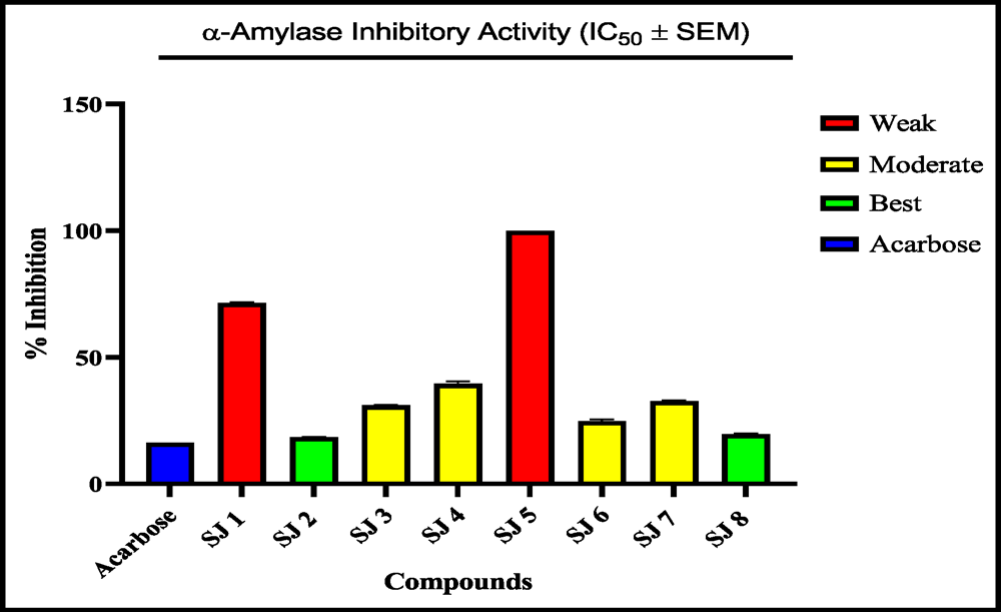
α‐Amylase inhibitory activity (% inhibition) of synthesized compounds (SJ1‐SJ8) compared with standard acarbose. SJ2 showed the highest inhibitory activity among all tested compounds.

SJ1, on the other hand, exhibited weak inhibition (71.56 ± 0.19 µM), suggesting a poor contact with the active site of the enzyme. Under the measured circumstances, SJ5 (IC_50_ >100 µM) was found to be inactive, indicating that inhibitory potential is greatly reduced when favorable substituents are absent. All things considered, the observed variations in activity across the synthesized compounds emphasize how crucial substitution patterns are in α‐amylase inhibition (as seen in Figure 3). The increased activity of SJ2 and SJ8 indicates the presence of important functional groups that enable more robust interactions with the enzyme, potentially via hydrophobic and hydrogen bonding interactions (as seen in Table 1).

**TABLE 1:** α‐Amylase inhibitory activity of indolo[2,1‐*b*]quinazoline‐6,12‐dione derivatives (SJ1‐SJ8).

Sr. no.	Compound	R^1^	R^2^	Structure	α‐Amylase inhibition IC_50_ ± SEM (µM)
**1**	**SJ1**	H	Cl	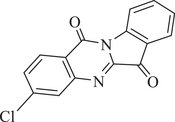	71.56 ± 0.19
**2**	**SJ2**	Br	Cl	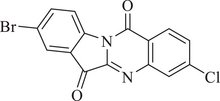	**18.49 ± 0.06**
**3**	**SJ3**	OCH3	Cl	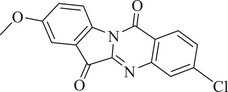	31.08 ± 0.12
**4**	**SJ4**	NO2	Cl	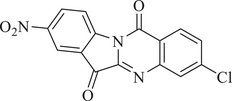	39.55 ± 0.80
**5**	**SJ5**	H	OH	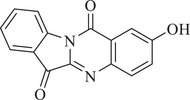	>100
**6**	**SJ6**	Br	OH	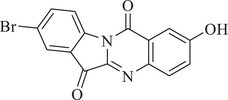	24.79 ± 0.60
**7**	**SJ7**	OCH3	OH	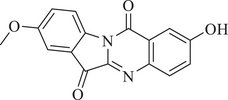	32.74 ± 0.20
**8**	**SJ8**	NO2	OH	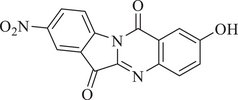	**19.67 ± 0.19**
**9**	**Acarbose**	–	–	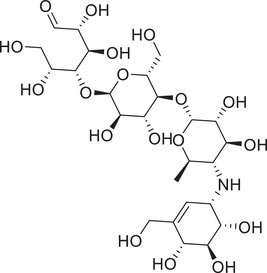	16.26 ± 0.02

### 2.3 Structure–Activity Relationships (SAR)

SAR study of the synthesized indolo[2,1‐*b*]quinazoline‐6,12‐dione derivatives (SJ1 ‐ SJ8) against α‐amylase inhibition emphasizes the importance of both positional substituent effects and the common core scaffold. As the basic pharmacophoric framework, the fused tetracyclic system provides planarity that enables π–π stacking interactions with TRP59, a crucial anchoring residue observed in the docked complexes. Furthermore, as crucial hydrogen bond acceptors, the dione functionalities at C‐6 and C‐12 help to stabilize interactions with residues including HIS299, HIS305, ASP300 and GLN63. Interestingly, all the derivatives had better docking scores than that of acarbose (─8.7 kcal/mol), indicating a good binding potential of the scaffold towards the α‐amylase active site.

The most important element influencing inhibitory efficacy was shown to be substitution at the R^1^ position (C‐8 of the indole ring). Substitution at C‐8 is necessary for considerable enzyme inhibition, as demonstrated by the greatly decreased or insignificant activity of compounds missing substitution at this position (R^1^ = H). As seen in SJ2 (IC_50_ = 18.49 µM), bromine (Br) exhibited the greatest activity among substituents within the chloro‐substituted (R^2^ = Cl) series. This is probably because of its large atomic size and polarizability, which enhance π‐stacking interactions and allow for more contacts with HIS305. Due to its electron‐donating properties and potential for weak C─H bonding interactions with ASP300, methoxy (O‐CH_3_) substitution produced modest activity. Strong electron‐withdrawing nitro (NO_2_) showed context‐dependent behavior, it decreased activity when paired with Cl at R^2^ but greatly increased potency when combined with OH (SJ8, IC_50_ = 19.67 µM), indicating a synergistic electron‐donating and electron‐withdrawing electronic effect that strengthen binding interactions.

The differences in activity can be explained using the concept of electronic effect, steric effect, polarizability, and hydrophobic complementarity. First, the presence of Br in SJ2 leads to increased polarizability, larger molecular volume, and hydrophobic effect, which are likely to favor dispersion and hydrophobic interactions inside the active site. The presence of the methoxy functional group in SJ3 will lead to hydrogen bond formation, as well as electron donation. However, this substituent will have less hydrophobic complementarity compared with Br. On the other hand, the strong electron withdrawing effect of NO_2_ leads to an increase in the polarity of the molecule, whose effect is dependent on the substituent present at position R^2^. The presence of OH along with NO_2_ greatly increases the activity when compared with the Cl analog.

**FIGURE 4:**
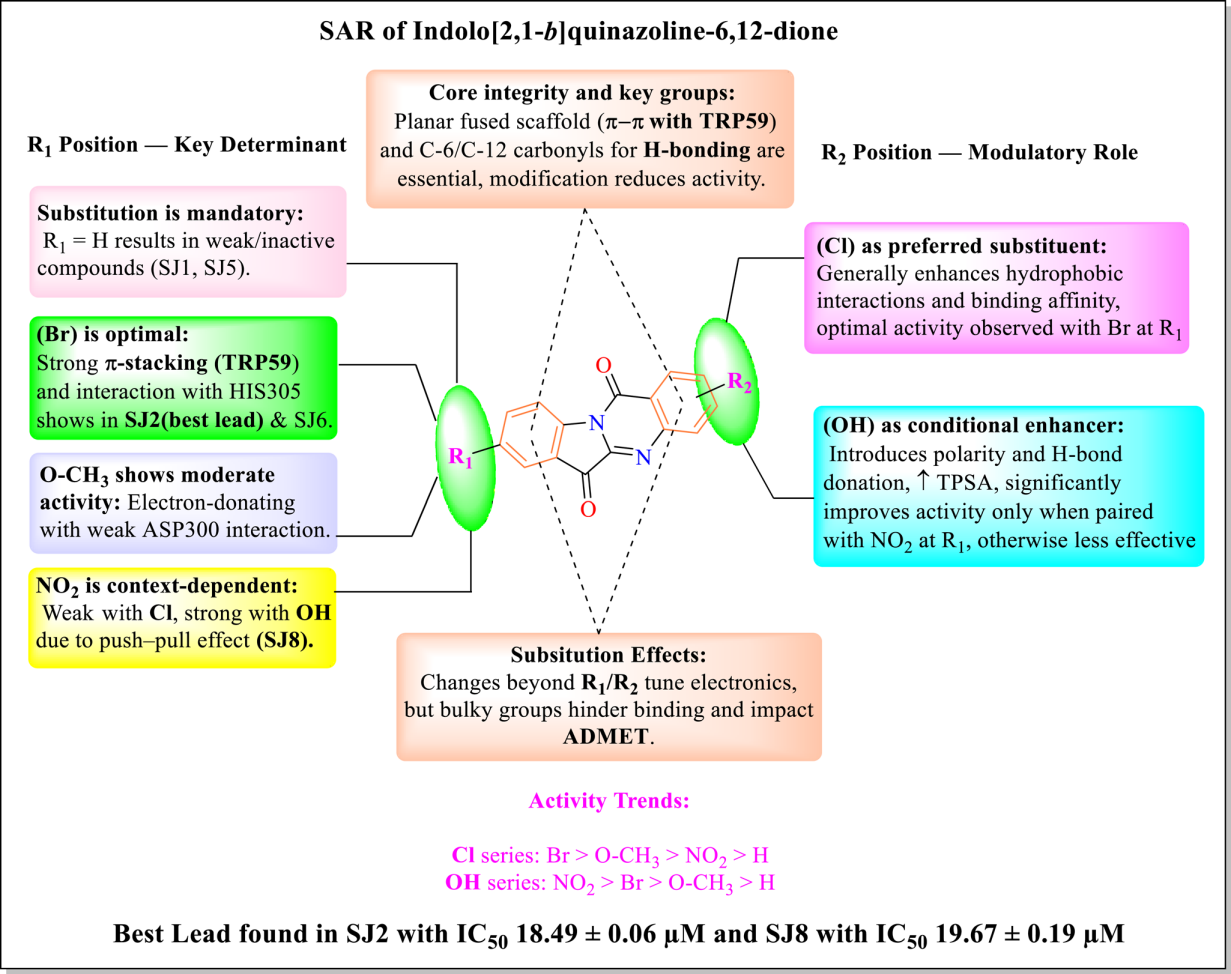
SAR profile of indolo[2,1‐*b*]quinazoline‐6,12‐dione derivatives highlighting the effects of R^1^/R^2^ substitutions on α‐amylase inhibitory activity.

Activity can be modified by adjusting the electronic, polarity, and hydrophobic properties of the molecule through the R^2^ position on the quinazoline ring. Because of its lipophilicity, which increases hydrophobic contacts inside the enzyme binding pocket and its mild electron‐withdrawing nature, chlorine (Cl) often exhibited higher activity. On the other hand, hydroxyl (OH) substitution enhanced polarity and added the potential to donate hydrogen bonds, but it was only beneficial when combined with a nitro group at R^1^, highlighting the significance of complementing electrical interactions. This interaction creates a distinct SAR trend in which Cl is generally favored and OH only becomes advantageous under certain substituent combinations (as seen in Figure 4).

Overall, substitution at R^1^ (C‐8) is necessary for activity, the combination of Br at R^1^ and Cl at R^2^ yields optimal inhibition, as shown by SJ2, which approaches the potency of acarbose. NO_2_ substitution at R^1^ requires OH at R^2^ for enhanced activity, preservation of the planar core scaffold is necessary for maintaining π–π stacking with TRP59. Increased polarity due to the presence of nitro analogs may affect hydrogen bonding and electrostatic interaction at the binding site of the α‐amylase, but since there is activity seen, this will depend on the identity of the R^2^ group. Additionally, whereas compounds like SJ4 and SJ6 showed toxicity issues despite having strong binding affinity, ADMET analysis revealed SJ2 as the best‐balanced candidate with positive safety features.

### 2.4 In Silico Studies

#### 2.4.1 Molecular Docking Studies

In order to gain insight into the structural properties of the inhibitory potential of α‐amylase, molecular docking analysis was performed to evaluate the binding properties of the selected derivatives against α‐amylase. Docking scores showed that all compounds had strong interactions with α‐amylase's catalytic pocket, suggesting promise as antidiabetic leads. Based on docking performance, the top eight compounds (SJ1‐SJ8) were chosen for synthesis and biological assessment. SJ2 had the greatest binding affinity (−9.2 kcal/mol), followed by SJ8 (−9.0 kcal/mol). The other derivatives had docking scores ranging from −8.8 to −8.9 kcal/mol. Docking research showed hydrogen bonding, π–π stacking and hydrophobic interactions with catalytically essential residues including TRP59, HIS305, ASP300 and TYR62, indicating the ligands' stable accommodation inside the enzyme active region (as shown in Tables 2 and 3). Compounds with bromine or hydroxyl substituents showed improved interaction patterns, indicating that electronic and hydrophobic factors have a substantial influence on binding affinity.

**TABLE 2:** Molecular docking binding energy and key interactions of designed ligands against α‐Amylase (PDB ID: 2QV4).

Compound	Binding energy (kcal/mol)	Key interactions with active site residues
REF	−8.7	Standard reference interaction profile
SJ1	−8.8	π‐cation (HIS201), π‐anion (ASP300), π‐alkyl (LYS200, ILE235, LEU162)
SJ2	−9.2	H‐bond (HIS305), π‐π stacking (TRP59), π‐alkyl (TYR62, HIS299)
SJ3	−8.9	H‐bond (HIS305), π‐π stacking (TRP59), C─H bond (ASP300), π‐alkyl (TYR62, HIS299)
SJ4	−8.9	H‐bond (HIS299), π‐π stacking (TRP59), C─H bond (HIS305)
SJ5	−8.8	π‐π stacking (TRP59), H‐bonds (GLN63, ASP300)
SJ6	−8.8	H‐bond (THR163), π‐donor H‐bond (TRP59), alkyl interactions (ILE235, LEU162, LEU165, HIS201)
SJ7	−8.8	H‐bonds (ARG195, HIS299, ASP197, TYR151), π‐anion (ASP300), π‐π T‐shaped (TYR62), π‐alkyl (LEU162)
SJ8	−9.0	H‐bond (ASP300), π‐π stacking (TRP59, TYR62)

**TABLE 3:** 2‐D and optimized 3‐D structures of the designed compounds and reference drug.

Compound ID	2D	3D
**Ref**	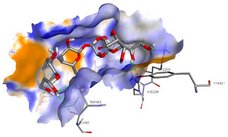	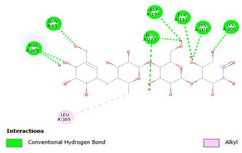
**SJ1**	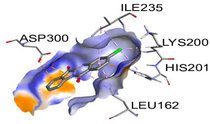	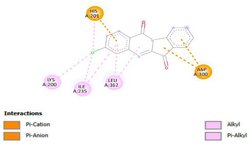
**SJ2**	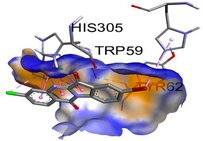	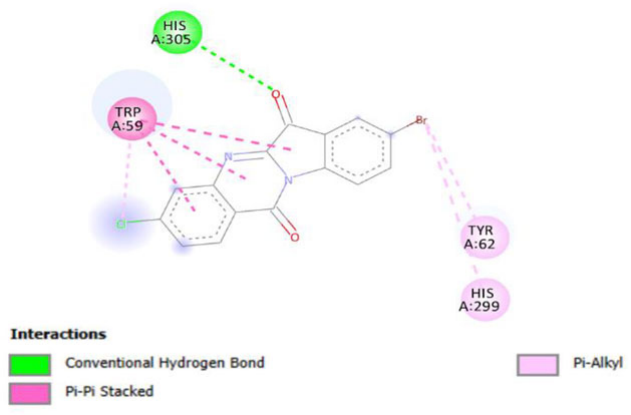
**SJ3**	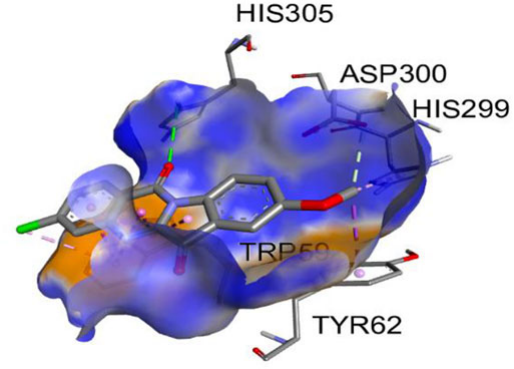	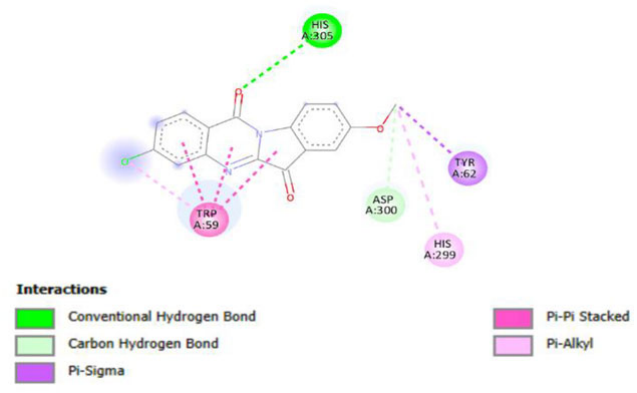
**SJ4**	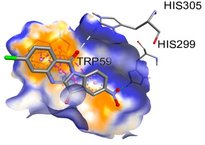	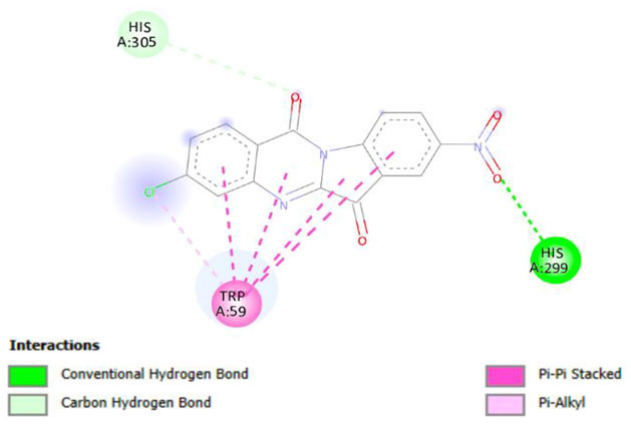
**SJ5**	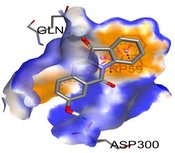	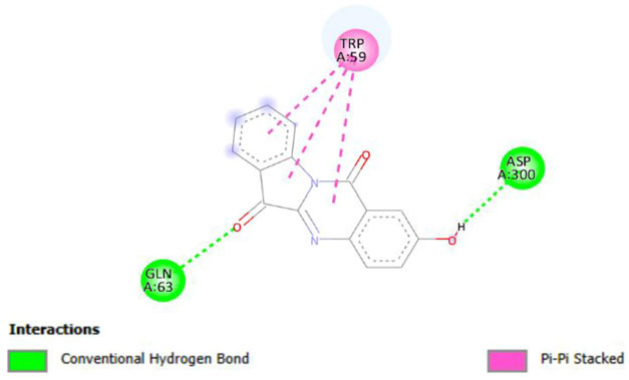
**SJ6**	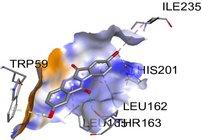	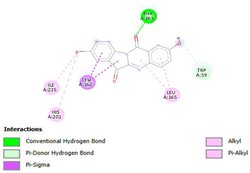
**SJ7**	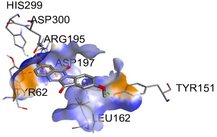	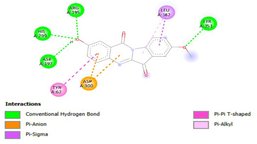
**SJ8**	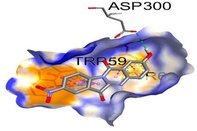	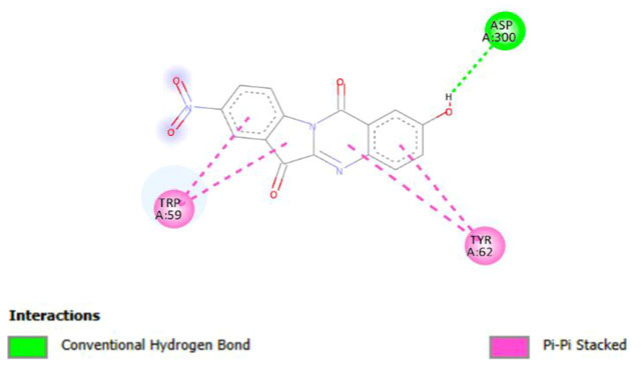

#### 2.4.2 Molecular Dynamics Studies

**FIGURE 5:**
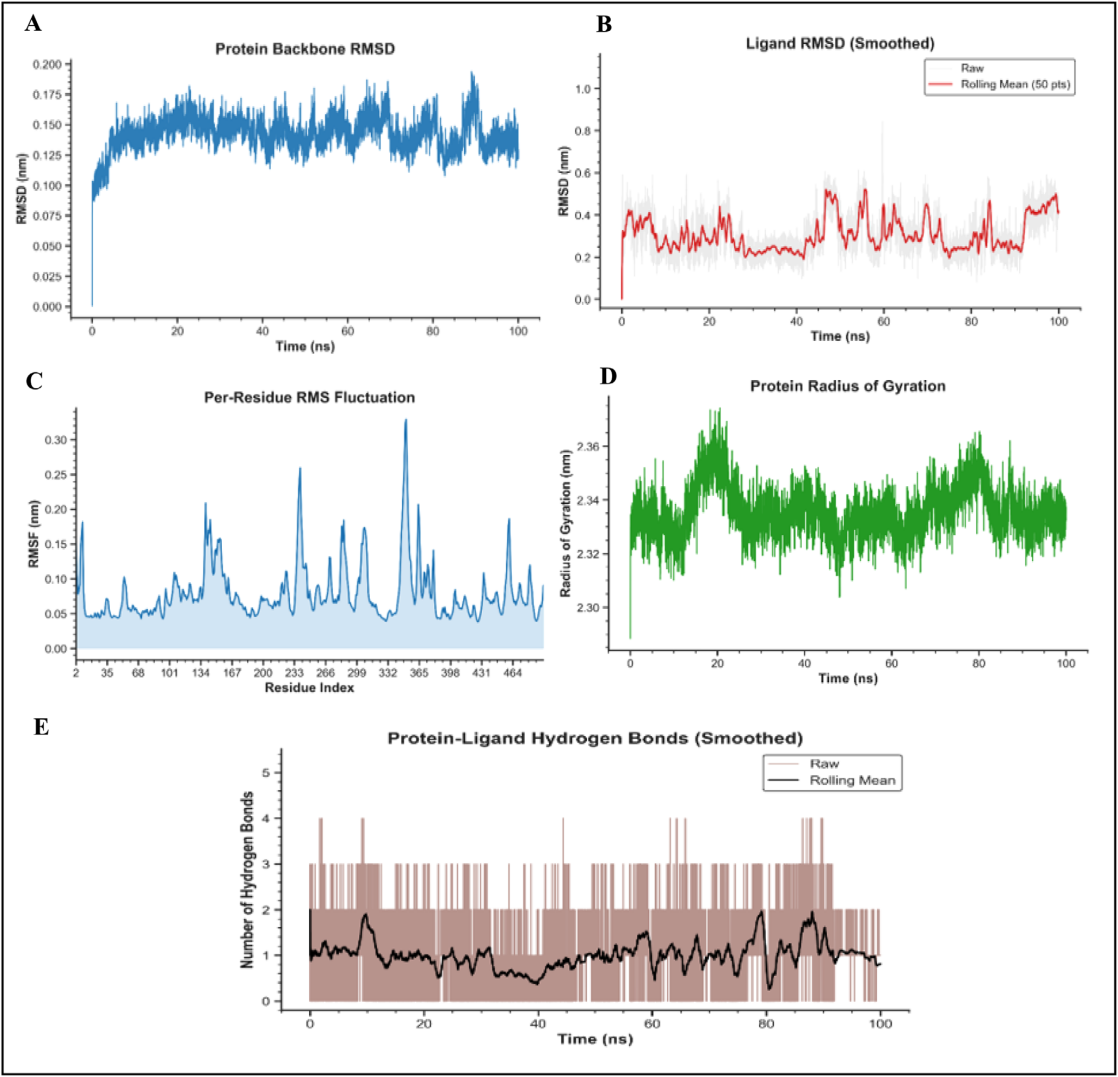
Molecular dynamics simulation analysis of the SJ2‐α‐Amylase complex showing (A) Protein backbone RMSD, (B) Ligand RMSD, (C) Residue‐wise RMSF, (D) Radius of gyration (Rg) and (E) Protein‐ligand hydrogen bond interactions during 100 ns simulation.

In order to gain more insights into the stability of the experimentally observed activity of SJ2, a 100 ns explicit solvent MD simulation of α‐amylase‐SJ2 complex was carried out to study the stability of the complex under physiological conditions. The protein backbone RMSD analysis showed rapid equilibration within the first 5–10 ns, after which the RMSD stabilized within a narrow range of 0.125–0.175 nm without significant structural drift or abrupt fluctuations, indicating the enzyme‐ligand complex's structural integrity and conformational stability throughout the simulation (as seen in Figure 5). The limited fluctuation range is typical of extremely stable protein‐ligand complexes, implying that SJ2 docked binding position is thermodynamically advantageous and dynamically accessible. Ligand RMSD measurements remained consistently below 0.30–0.35 nm throughout the whole 100 ns run period with no evidence of any ligand dissociation. The ligand RMSD calculation using a smooth rolling average of 50 points confirmed the presence of an occupied active site. Important catalytic‐site residues such as TRP58, TRP59, HIS305, GLN63 and TYR62, showed little variations (< 0.10 nm) according to residue‐wise RMSF analysis, indicating that the active‐site cavity remained stiff and conformationally stable during ligand interaction (as seen in Figure 5). Overlaid RMSD diagram of protein backbone and S2 ligand has been depicted in Figure S9. Solvent accessible surface area (SASA) of alpha‐amylase has been presented in Figure S10. Figure S11 confirms that α‐Helical and β‐strands are maintained throughout the simulation studies.

Conversely, only distal solvent‐exposed loop regions, which are not directly engaged in catalytic interactions showed more flexibility. All of these results show that SJ2 stabilizes the α‐amylase binding pocket without causing significant structural changes. Additionally, the radius of gyration (Rg), which fluctuated only between 2.30 and 2.37 nm with an average value of ∼2.34 nm, remained extremely stable throughout the simulation, confirming the preservation of the α‐amylase compact globular fold and the lack of unfolding or domain separation events during ligand binding (as seen in Figure 5). Further evidence of SJ2 robust and long‐lasting stabilization inside the α‐amylase catalytic cleft during the simulation came from protein‐ligand interaction studies. Throughout the 100 ns simulation SJ2 continuously maintained one to three hydrogen bonds with active‐site residues, according to hydrogen bond trajectory analysis with no extended periods of total contact loss. The ligand's persistent polar anchoring inside the binding cavity was confirmed by the smoothed rolling mean profile which stayed steady at around 1.0–1.5 hydrogen bonds. One hydrogen bond (∼51% of simulation frames) was the most prevalent interaction state, followed by two hydrogen bonds (∼18%), according to hydrogen bond frequency analysis, showing persistent intermolecular stabilization (as seen in Figure 5).

While hydrophobic and aromatic interactions with TRP58 and TRP59 further strengthened ligand binding, persistent hydrogen bonding interactions involving the ligand carbonyl groups with residues like HIS305 and GLN63 greatly contributed to the complex's directional stability. The exceptional stability of the α‐amylase–SJ2 complex exhibited throughout the molecular‐dynamics trajectory may be explained by the preponderance of these long‐lasting hydrophobic, π–π stacking and hydrogen‐bonding contacts, which are perfectly compatible with the MM‐PBSA energy decomposition data.

Figure S12 presents protein‐ligand hydrogen bond analysis during simulation which showed 1‐2 Hydrogen bonds as the dominant states. Protein ligand contacts have been depicted in Figure S13.

#### 2.4.3 MM‐PBSA Binding Free Energy Components

**FIGURE 6:**
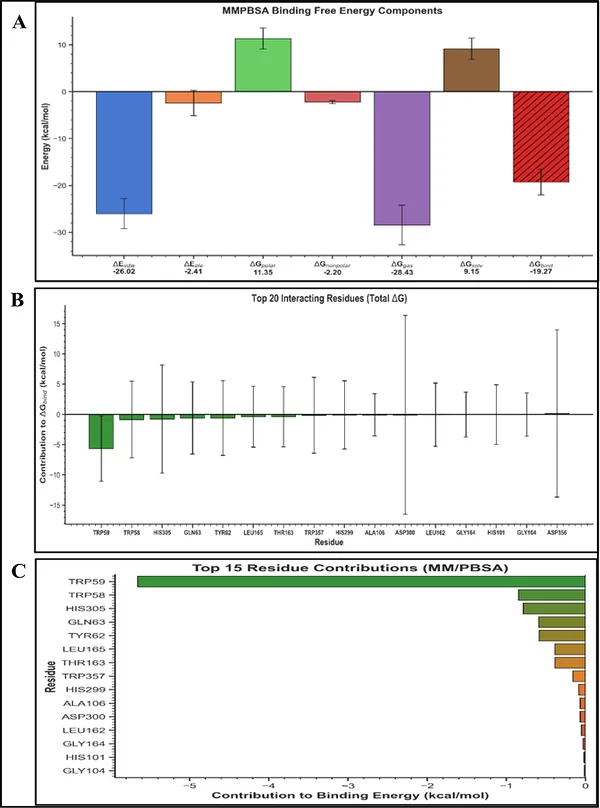
MM/PBSA binding free energy analysis of the SJ2‐α‐Amylase complex showing (A) Binding free energy components, (B) Top interacting residues contributing to total binding energy and (C) Top 15 residue‐wise energy contributions during the simulation.

The MM‐PBSA binding free energy study of the α‐amylase‐SJ2 complex indicated thermodynamically favorable and extremely stable ligand binding, with a net binding free energy of Δ*G*_bind_ = −19.27 kcal/mol, indicating spontaneous complex formation and substantial inhibition potential. Van der Waals interactions were shown to be the major stabilizing force (Δ*E*_VdW_ = −26.02 kcal/mol), indicating that hydrophobic and π–π stacking interactions are the primary mechanisms influencing ligand recognition inside the α‐amylase active site. This large hydrophobic contribution is consistent with SJ2 highly conjugated planar indoloquinazolinedione scaffold which effectively interacts with the catalytic pocket's aromatic residues. The binding environment was mostly non‐polar, resulting in a minor electrostatic contribution (Δ*E*_ele_ = −2.41 kcal/mol) (as seen in Figure 6). However, directed hydrogen bonding interactions with residues like HIS305 and GLN63 added stability. The polar solvation energy contributed unfavorably (+11.35 kcal/mol) due to desolvation penalties associated with ligand binding. However, this energetic cost was compensated by favorable non‐polar solvation energy (Δ*G*_nonpolar_ = −2.19 kcal/mol) and strong van der Waals attraction, resulting in a highly negative and favorable total binding free energy (as seen in Figure 6). SJ2 computed Δ*G*_bind_ value is within the range of high‐affinity inhibitors, confirming its α‐amylase inhibitory efficacy and making it a viable antidiabetic lead candidate.

Per‐residue MM‐PBSA free energy decomposition revealed the key pharmacophoric residues that stabilize the α‐amylase‐SJ2 complex. TRP59 contributed the most positively (∼ −5.5 kcal/mol), followed by TRP58 (∼ −4.0 kcal/mol) and HIS305 (∼ −3.5 kcal/mol), accounting for the bulk of total binding free energy. GLN63 (∼ −2.5 kcal/mol) and TYR62 (∼ −2.0 kcal/mol) provided further stabilization, whereas the remaining residues had moderately favorable interactions ranging from −0.5 to −1.5 kcal/mol. TRP59 and TRP58 play a significant role, highlighting the significance of aromatic π–π stacking interactions between SJ2 electron‐deficient fused tetracyclic core and the indole side chains of tryptophan residues at the active site. HIS305 contributed through both hydrogen bonding and hydrophobic contacts, notably in the ligand's bromine‐substituted region, whereas GLN63 stabilized the complex by directed hydrogen bonding with the carbonyl functions of the quinazolinedione molecule (as seen in Figure 6).

#### 2.4.4 Conformational Dynamics and Free Energy Analysis

**FIGURE 7:**
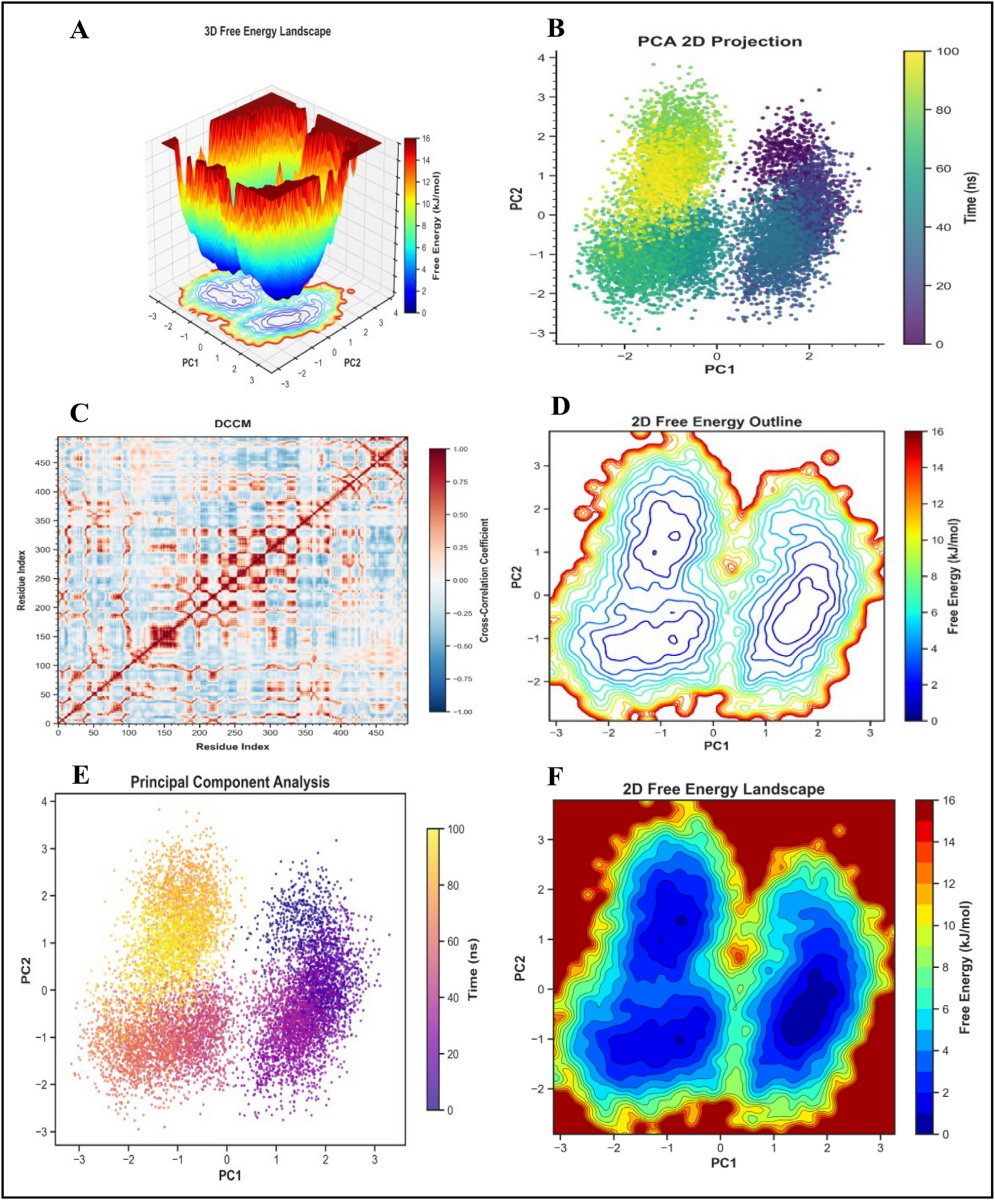
PCA, free energy landscape and DCCM analyses of the α‐amylase‐SJ2 complex during 100 ns MD simulation. (A) 3D free energy landscape, (B, E) time‐colored PCA projections showing conformational transition, (C) DCCM plot showing correlated and anti‐correlated residue motions and (D, F) contour and filled 2D free energy landscapes revealing two stable metastable conformational states.

During the 100 ns MD simulation, the conformational dynamics and thermodynamic stability of the α‐amylase‐SJ2 complex were examined using principal component analysis (PCA), free energy landscape (FEL) and dynamic cross‐correlation matrix (DCCM) studies. Two separate conformational clusters were primarily separated along the PC1 axis in the time‐colored PCA 2D projections. Early simulation frames (purple/blue) occupied positive PC1 regions and later stages (green/yellow) gradually shifted toward negative PC1 values, indicating conformational adaptation from the initial binding state to a more equilibrated and stable conformation. Throughout the simulation, the clustering pattern verified that conformational sampling with limited structural variations converged. The complex's excellent conformational stability was further shown by the 2‐D FEL analysis. A compact and thermodynamically stable dominant conformation with little unfolding or instability was indicated by the RMSD versus radius of gyration (Rg) FEL, which showed a single deep global energy basin centered at Rg ≈ 2.32–2.34 nm and RMSD ≈ 1.25–1.5 Å, corresponding to the lowest free energy state (∼0 kJ/mol). Furthermore, the PC1‐PC2 FEL showed two low‐energy minima at roughly (PC1 = ‐2, PC2 = ‐1) and (PC1 = 1, PC2 = ‐1), separated by a tiny energy barrier of ∼4–6 kJ/mol, indicating the presence of two metastable conformational states that can quickly interconvert under physiological conditions. Strong resistance to ligand dissociation and persistent SJ2 binding inside the α‐amylase active site were shown by the contour‐line and 3D FEL representations, which further emphasized the transition paths and deep energy wells encircled by steep energetic barriers. While maintaining important intermolecular contacts, these conformational changes are probably linked to a minor reorientation of the asymmetric halogenated SJ2 scaffold inside the binding cavity. Additionally, large positively correlated motions among residues inside α‐helices and β‐strands were shown by DCCM analysis, indicating coordinated rigid‐body domain movements, whereas anti‐correlated areas showed hinge‐like flexibility that contributed to intrinsic enzyme dynamics. The dynamic correlation patterns obtained indicate that SJ2 binding stabilizes the global motion network of α‐amylase and controls inter‐domain communication. The α‐amylase‐SJ2 complex sustains excellent thermodynamic stability, structural compactness, regulated conformational flexibility and stable active‐site interactions throughout the MD simulation, as confirmed by the PCA, FEL and DCCM studies taken together (as seen in Figure 7).

#### 2.4.5 DFT Studies

**FIGURE 8:**
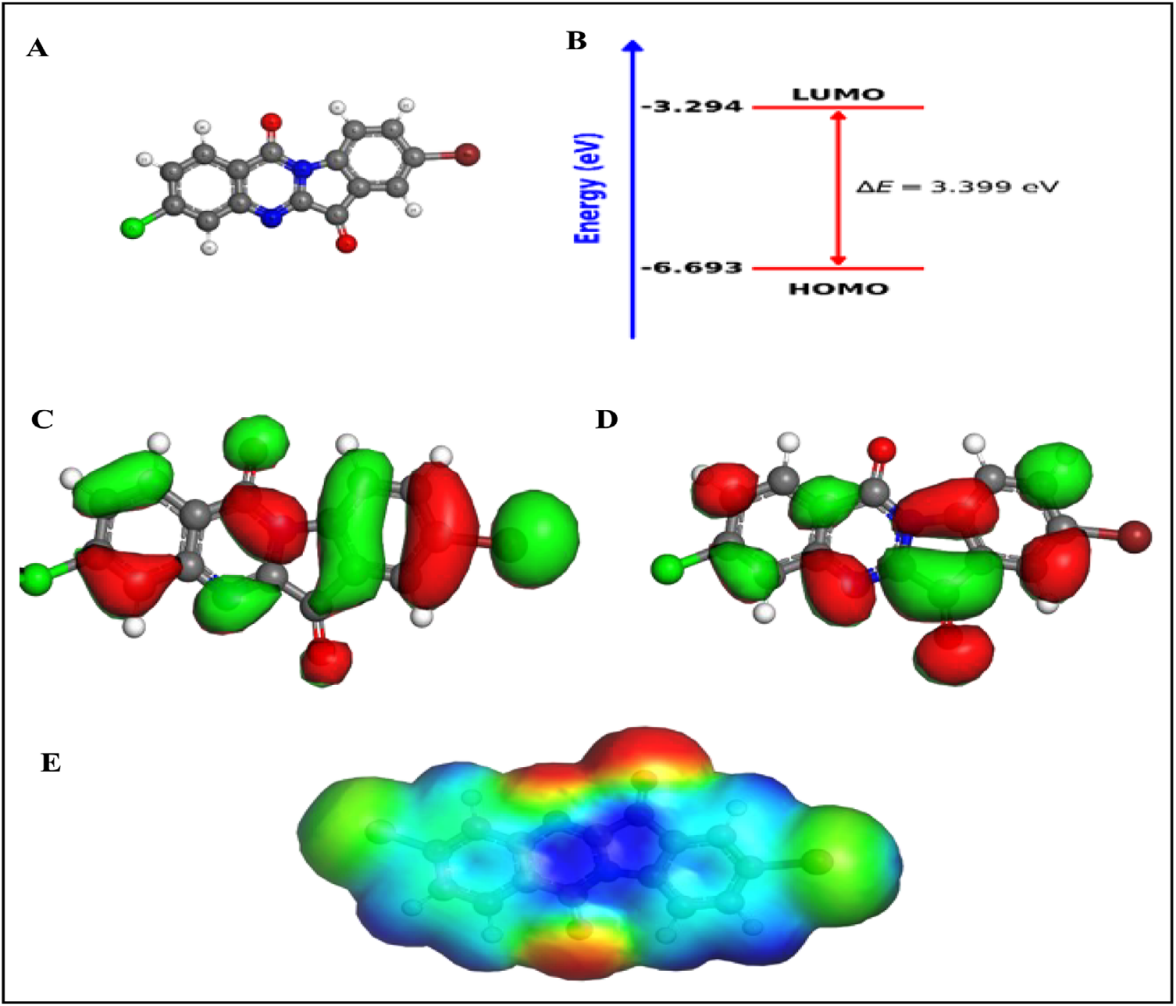
Density functional theory (DFT) analysis of SJ2 showing (A) Optimized molecular geometry, (B) HOMO–LUMO energy gap, (C) HOMO electron density distribution, (D) LUMO electron density distribution and (E) molecular electrostatic potential (MEP) surface map.

DFT analysis of SJ2 at the B3LYP/6‐311G level revealed a HOMO energy of −6.693 eV and a LUMO energy of −3.294 eV, producing a moderate HOMO‐LUMO energy gap (ΔE) of 3.399 Ev (as seen in Figure 8). This indicates a chemically reactive and charge‐transfer‐capable scaffold with favorable biological interaction potential. Frontier molecular orbital analysis revealed extensive HOMO delocalization across the fused indole‐quinazoline‐dione π‐system, along with contributions from nitrogen lone pairs, resulting in a broad electron‐rich aromatic surface capable of strong π–π stacking interactions with active‐site residues TRP59 and TRP58 of α‐amylase. In contrast, the LUMO was largely concentrated over the quinazolinedione carbonyl groups at C‐6 and C‐12, as well as partly over the Br and Cl substituents, indicating that these areas are the primary electrophilic and hydrogen‐bond‐accepting sites engaged in interactions with HIS305 and GLN63. Molecular electrostatic potential (MEP) mapping within the range of −0.04 to +0.04 a.u. confirmed this electronic complementarity. The carbonyl oxygen atoms appeared strongly electron‐rich red regions suitable for hydrogen bonding, while the fused aromatic core displayed an electron‐deficient blue π‐surface favorable for aromatic stacking. The Br and Cl substituents had intermediate green areas, indicating polarizability‐driven halogen interactions in the binding pocket (as seen in Figure 8). SJ2 has a high electrophilicity index (*ω* = 7.34 eV) and moderate chemical hardness (*η* = 1.700 eV), indicating effective interaction with nucleophilic residues and soft π‐electron systems. This supports the experimentally reported van der Waals‐ and π‐stacking‐dominated binding profiles. The HOMO/LUMO and MEP studies support SJ2 high‐affinity interaction with α‐amylase, resulting in a favorable MM‐PBSA binding energy and long‐term molecular dynamics stability (as seen in Figure 8).

#### 2.4.6 ADMET Studies

In silico ADMET profiling revealed that all compounds have good drug‐like features, such as acceptable molecular weight, moderate lipophilicity, high gastrointestinal absorption and adherence to Lipinski's rule of five. Most derivatives, especially SJ2, SJ3, SJ6, and SJ7, showed low anticipated toxicity, no hERG‐I inhibition and a low risk of hepatotoxicity, indicating potential pharmacokinetic and safety properties. The docking and ADMET results indicate that the synthesized indoloquinazoline derivatives are potential α‐amylase inhibitors for future antidiabetic medication development (as seen in Table 4). More details are given in Tables S1 and S2.

**TABLE 4:** In silico ADME and toxicity profiling of designed indolo[2,1‐*b*]quinazoline derivatives.

Compound	MW (g/mol)	TPSA (Å^2^)	iLogP	Water solubility	GI absorption	BBB permeability	AMES toxicity	hERG I	Hepatotoxicity
SJ1	282.68	51.96	2.18	Soluble	High	Yes	Yes	No	No
SJ2	361.58	51.96	2.48	Moderately soluble	High	Yes	No	No	No
SJ3	312.71	61.19	2.45	Soluble	High	Yes	No	No	No
SJ4	327.68	97.78	1.68	Soluble	High	No	Yes	No	Yes
SJ5	264.24	72.19	1.55	Soluble	High	Yes	Yes	No	No
SJ6	343.13	72.19	1.86	Moderately soluble	High	Yes	No	No	No
SJ7	294.26	81.42	1.81	Soluble	High	No	No	No	No
SJ8	309.23	118.01	1.08	Soluble	High	No	Yes	No	No

## 3 Conclusion

The study showed that indolo[2,1‐b]quinazoline‐6,12‐dione derivatives are promising in vitro α‐amylase inhibitors and may be useful leads in the development of new antidiabetic drugs. SJ2 emerged as the most promising lead chemical among the synthesized derivatives due to its outstanding inhibitory action, attractive docking score, stable molecular dynamics profile and favorable MM‐PBSA binding free energy. Computational investigations suggest that SJ2 shows favorable binding within the α‐amylase active site via π–π stacking, hydrophobic interactions, and hydrogen bonding with critical residues such TRP59, TRP58, HIS305, GLN63, and TYR62. Molecular dynamics simulations revealed sustained binding stability over 100 ns without causing structural instability of the enzyme. The dominating contribution of van der Waals interactions resulted in hydrophobic and aromatic contacts being the primary driving factors influencing ligand recognition and stability. DFT and MEP investigations provided insights into the electronic features of SJ2 that may contribute to its biological interactions, revealing electron‐rich carbonyl areas capable of accepting hydrogen bonds and a delocalized aromatic π‐system suitable for stacking interactions. Moreover, in silico ADMET analysis revealed favorable predicted pharmacokinetic and toxicity properties of SJ2, providing support for further studies of this compound; however, further experimental testing is required for verification of these predictions.

Overall, the results indicate that the fused indoloquinazolinedione framework is a promising pharmacophore for the development of α‐amylase inhibitory leads with potential antidiabetic relevance. However, proposed molecular interactions were based on computational analyzes and not experimentally validated, and the biological evaluation here was restricted to in vitro α‐amylase inhibition. Therefore, SJ2 is a promising preliminary lead, but not a validated antidiabetic therapeutic candidate. Mechanistic, cell‐based and in vivo studies are needed to further evaluate its biological efficacy, safety and therapeutic potential.

Further experimental evaluation of the synthesized derivatives against α‐glucosidase should be carried out in future studies to determine whether the observed inhibitory activity against α‐amylase extends to the complementary carbohydrate digesting enzyme α‐glucosidase. Comparative α‐amylase/α‐glucosidase profiling, enzyme kinetic and inhibition‐mechanism studies, and appropriate cell‐based and in vivo investigations will be important for establishing the broader antidiabetic potential of the indoloquinazolinedione scaffold. Such studies may also provide further structure–activity relationship information, and they may help to determine the solubility, oral bioavailability, pharmacokinetic behavior, selectivity, gastrointestinal tolerability, safety and therapeutic potential of SJ2 and related derivatives.

## 4 Experimental Section

### 4.1 General

Anthranilic acid (1 Equivalent) derivatives (**1**) were initially treated with few drops thionyl chloride (SOCl_2_) in dry toluene (10–15 mL) under reflux conditions (100°C–110°C), resulting in the conversion of the carboxylic acid group into the corresponding 2‐aminobenzoyl chloride intermediate (**2**) while simultaneously eliminating sulfur dioxide (SO_2_) and hydrogen chloride (HCl). Subsequently, (1 Equivalent) substituted indoline‐2,3‐dione (isatin) derivatives (**3**) were added to the reaction mixture, where the ortho‐amino group of 2‐aminobenzoyl chloride attacked the electrophilic C‐3 carbonyl carbon of isatin, resulting in the formation of the amide intermediate 1‐(2‐aminobenzoyl)indoline‐2,3‐dione (**4**) and the loss of HCl. After further refluxing, the intermediate underwent intramolecular cyclization and dehydration, yielding the fused indolo[2,1‐*b*]quinazoline‐6,12‐dione derivatives (SJ1‐SJ8) (**5**). TLC was used to monitor reaction completion before cooling the mixture to room temperature and extracting the solvent under decreased pressure. To breakdown leftover thionyl chloride and enable precipitation of the crude product, the residue was put onto crushed ice while being stirred constantly [40]. The solid was vacuum‐filtered, rinsed completely with cold water to remove acidic and inorganic impurities, then washed again with cold ethanol to remove unreacted starting ingredients and non‐polar impurities. Finally, the crude product was refined by recrystallization with an appropriate organic solvent to provide the pure substituted indoloquinazoline‐dione derivative as a stable crystalline solid [41]. The spectral characterization data for compounds SJ1‐SJ8 have been presented in Figures S1–S8.

#### 4.1.1 (SJ1)3‐chloroindolo[2,1‐b]quinazoline‐6,12‐dione

It was obtained as solid pale crystals; yeild 65%; M. pt. 320‐325°C, FT‐IR (cm^−^^1^): 1776.9, 1667.5 (C═O, quinazoline‐6,12‐dione), 1565.3, 1513.5 (Ar C═C/C═N), 1317.6‐1047.2 (C‐N, heterocyclic framework), 759.4, 674.1 (Ar‐Cl), 1H NMR (500 MHz, DMSO‐*d*_6_) δ 8.18 (d, *J* = 7.6 Hz, 2H, H‐1/H‐2), 8.11 (dd, *J* = 6.9, 1.3 Hz, 1H, H‐7 or H‐10), 7.90 (dd, *J* = 6.6, 1.2 Hz, 1H, H‐10 or H‐7), 7.80 (d, *J* = 2.0 Hz, 1H, H‐4), 7.69 (ddd, *J* = 7.7, 6.6, 1.3 Hz, 1H, H‐8 or H‐9), 7.54‐7.49 (m, 1H, H‐9 or H‐8), 13C NMR (126 MHz, DMSO) δ 181.19 (C‐18, indole lactam C═O), 155.62 (C‐19, quinazoline lactam C═O), 149.82 (C‐8/C‐11, quaternary C‐N), 146.37 (C‐7/C‐13, quaternary C‐N), 143.79 (C‐16, C‐Cl), 137.30 (C‐10/C‐12, fused quaternary C), 134.10 (C‐9/C‐17, fused quaternary C), 127.60 (C‐14/C‐3, Ar‐CH), 127.43 (C‐11/C‐2, Ar‐CH), 126.66 (C‐15/C‐1, Ar‐CH), 126.26 (C‐2/C‐11, Ar‐CH), 122.83 (C‐1/C‐15, Ar‐CH), 120.31 (C‐4, Ar‐CH, ortho to Cl), 118.29 (C‐5, Ar‐CH), HRMS (ESI^+^): *m/z* calculated for C_15_H_8_ClN_3_O_2_ [M+H]^+^: 283.00; found: 283.0363; [M+2]^+^: 285.0380.

#### 4.1.2 (SJ2)8‐bromo‐3‐chloroindolo[2,1‐b]quinazoline‐6,12‐dione

It was obtained as solid dark yellow crystals; yeild 72%; M.pt. 318‐322°C, FT‐IR (cm^−^^1^): 1752.35, 1707.51 (C═O, quinazoline‐6,12‐dione), 1613.59 (Ar C═C/C═N), 1278.84‐1121.06 (C‐N), 745.51, 672.60 (Ar‐Cl), 625.54 (Ar‐Br), ^1^H NMR (500 MHz, DMSO‐d_6_) δ 8.14‐8.06 (m, 2H, H‐1/H‐2), 7.84‐7.74 (m, 2H, H‐4/H‐7), 7.66 (d, *J* = 8.3 Hz, 1H, H‐10), 7.41 (dd, *J* = 7.7, 2.0 Hz, 1H, H‐9), 13C NMR (126 MHz, DMSO) δ 182.95 (C‐18, indole lactam C═O), 158.75 (C‐19, quinazoline lactam C═O), 149.30 (C‐7/C‐13, quaternary C‐N), 145.57 (C‐10/C‐11, quaternary C‐N), 139.83 (C‐16, C‐Cl), 139.69 (C‐4/C‐5, fused quaternary C), 133.39 (C‐1, C‐Br), 127.38 (C‐14/C‐3, Ar‐CH), 126.66 (C‐15/C‐2, Ar‐CH), 123.70 (C‐6/C‐17, Ar‐CH), 121.15 (C‐2/C‐15, Ar‐CH), 119.31 (C‐17/C‐14, Ar‐CH), 116.44 (C‐3, Ar‐CH, ortho to Br), 114.06 (C‐6, Ar‐CH), HRMS (ESI^+^): *m/z* calculated for C_15_H_6_BrClN_2_O_2_ [M+H]^+^: 359.93, found: 360.0525, [M+2]^+^: 362.0505, [M+4]^+^: 364.0525.

#### 4.1.3 (SJ3)3‐chloro‐8‐methoxyindolo[2,1‐b]quinazoline‐6,12‐dione

It was obtained as solid reddish brown crystals; yeild 69%; M.pt. 330‐335°C, FT‐IR (cm^−^^1^): 1655.45 (C═O), 1511.53 (Ar C═C/C═N), 1236.17, 1031.36 (Ar O‐CH_3_), 678.57 (Ar‐Cl), 963.71, 833.26 (aromatic substitution), 1H NMR (500 MHz, DMSO‐*d*6) δ 8.14 (d, *J* = 8.0 Hz, 1H), 7.68 (dd, *J* = 5.1, 3.1 Hz, 2H), 7.56 (d, *J* = 2.9 Hz, 1H), 7.19 (dd, *J* = 8.5, 2.7 Hz, 1H), 6.52 (dd, *J* = 8.6, 2.1 Hz, 1H), 3.75 (s, 3H), 13C NMR (126 MHz, DMSO) 184.56 (C‐9, lactam C═O), 168.74 (C‐10, lactam C═O), 159.44 (C‐2, C═N/C‐O, quaternary), 155.22 (C‐1, Ar‐C‐OCH_3_), 152.30 (C‐8, quaternary C‐N), 144.56 (C‐4, quaternary aromatic C), 138.12 (C‐7, quaternary aromatic C), 132.98 (C‐16, Ar‐C‐Cl), 124.77 (C‐14, Ar‐CH), 117.99 (C‐15, Ar‐CH), 114.97 (C‐17, Ar‐CH), 114.43 (C‐6, Ar‐CH), 113.12 (C‐5, Ar‐CH), 108.69 (C‐3, Ar‐CH), 108.49 (C‐12, Ar‐CH), 55.69 (C‐22, OCH_3_), HRMS (ESI^+^): *m/z* calculated for C_16_H_9_ClN_2_O_3_ [M+H]^+^: 313.0380, found: 313.0498, [M+2]^+^: 315.0485.

#### 4.1.4 (SJ4)3‐chloro‐8‐nitroindolo[2,1‐b]quinazoline‐6,12‐dione

It was obtained as solid dark‐yellow crystals; yeild 61%; M.pt. 315‐320°C, FT‐IR (cm^−^^1^): 1766.95, 1701.14 (C═O, quinazoline‐6,12‐dione), 1608.03 (Ar C═C/C═N), 1518.47 (NO_2_ asymmetrical), 1332.52 (NO_2_ symmetrical), 1237.88‐1053.60 (C‐N), 675.64 (Ar‐Cl), ^1^H NMR (500 MHz, DMSO‐*d*_6_) δ 8.96 (dd, *J* = 8.8, 2.8 Hz, 1H, H‐2), 8.69 (d, *J* = 2.3 Hz, 1H, H‐4), 8.21 (d, *J* = 7.6 Hz, 1H, H‐10), 8.13 (d, *J* = 8.7 Hz, 1H, H‐1), 7.86 (d, *J* = 2.0 Hz, 1H, H‐7), 7.48 (dd, *J* = 8.0, 2.2 Hz, 1H, H‐9), 13C NMR (126 MHz, DMSO) 182.36 (C‐9, lactam C═O), 181.65 (C‐10, lactam C═O), 159.87 (C‐8, C═N), 159.31 (C‐11, quaternary C‐N), 155.18 (C‐4, quaternary C‐N), 147.51 (C‐1, Ar‐C‐NO_2_), 143.57 (C‐7, quaternary aromatic C), 142.61 (C‐12, quaternary aromatic C), 133.06 (C‐16, Ar‐C‐Cl), 127.29 (C‐2, Ar‐CH), 123.29 (C‐14, Ar‐CH), 119.57 (C‐3, Ar‐CH), 118.14 (C‐5, Ar‐CH), 112.50 (C‐6, Ar‐CH), HRMS (ESI^+^): *m/z* calculated for C_15_H_7_ClN_4_O_4_ [M+H]^+^: 327.00, found: 327.0349, [M+2]^+^: 329.0150.

#### 4.1.5 (SJ5)2‐hydroxyindolo[2,1‐b]quinazoline‐6,12‐dione

It was obtained as solid brown crystals; yeild 65%; M.pt. 325‐330°C, FT‐IR (cm^−^^1^): 3217.51 (OH), 1729.04, 1606.56 (C═O, quinazoline‐6,12‐dione), 1493.36 (Ar C═C/C═N), 1303.66, 1208.30 (C‐N/C‐O), 764.62, 684.65 (aromatic C‐H), 1H NMR (500 MHz, DMSO‐*d*6) δ 9.69 (s, 1H), 8.25 (dd, *J* = 7.1, 1.4 Hz, 1H), 7.98 (dd, *J* = 6.5, 1.3 Hz, 1H), 7.83 (ddd, *J* = 7.6, 6.6, 1.3 Hz, 1H), 7.62 (d, *J* = 9.0 Hz, 1H), 7.65 ‐ 7.54 (m, 2H), 7.14 (dd, *J* = 9.0, 2.0 Hz, 1H), 13C NMR (126 MHz, DMSO) 182.26 (C‐9, lactam C═O), 169.21 (C‐10, lactam C═O), 158.97 (C‐15, Ar‐C‐OH), 152.14 (C‐11, quaternary C‐N), 146.92 (C‐4, quaternary C‐N), 146.71 (C‐7, quaternary aromatic C), 138.26 (C‐12, quaternary aromatic C), 137.43 (C‐8, fused quaternary C), 124.57 (C‐2, Ar‐CH), 122.87 (C‐14, Ar‐CH), 122.65 (C‐17, Ar‐CH), 117.76 (C‐3, Ar‐CH), 115.18 (C‐5, Ar‐CH), 112.09 (C‐6, Ar‐CH), HRMS (ESI^+^): *m/z* calculated for C_15_H_8_N_2_O_3_ [M+H]^+^: 263.0452, found: 264.0487.

#### 4.1.6 (SJ6)8‐bromo‐2‐hydroxyindolo[2,1‐b]quinazoline‐6,12‐dione

It was obtained as solid dark yellow crystals; yeild 67%; M.pt. 315‐320°C, FT‐IR (cm^−^^1^): 3216.97 (OH), 1747.92, 1706.29 (C═O, quinazoline‐6,12‐dione), 1609.19, 1504.16 (Ar C═C/C═N), 1307.11, 1207.11 (C‐N/C‐O), 671.95 (Ar‐Br), 1H NMR (500 MHz, DMSO‐*d*6) δ 9.29 (s, 1H), 8.11 (d, *J* = 3.6 Hz, 1H), 7.74 (dd, *J* = 8.4, 2.1 Hz, 1H), 7.61 ‐ 7.56 (m, 2H), 7.55 (d, *J* = 2.3 Hz, 1H), 7.11 (dd, *J* = 9.0, 2.0 Hz, 1H), 13C NMR (126 MHz, DMSO) 183.07 (C‐9, lactam C═O), 162.80 (C‐10, lactam C═O), 158.86 (C‐15, Ar‐C‐OH), 155.03 (C‐11, quaternary C‐N), 149.46 (C‐4, quaternary C‐N), 139.92 (C‐7, quaternary aromatic C), 138.68 (C‐12, quaternary aromatic C), 134.01 (C‐1, Ar‐C‐Br), 128.09 (C‐2, Ar‐CH), 126.78 (C‐14, Ar‐CH), 123.94 (C‐17, Ar‐CH), 121.83 (C‐3, Ar‐CH), 119.46 (C‐5, Ar‐CH), 114.16 (C‐6, Ar‐CH), HRMS (ESI^+^): *m/z* calculated for C_15_H_8_BrN_2_O_3_ [M+H]^+^: 342.9712, found: 342.9788, [M+2+H]^+^: 344.9756.

#### 4.1.7 (SJ7)2‐hydroxy‐8‐methoxyindolo[2,1‐b]quinazoline‐6,12‐dione

It was obtained as solid redish brown crystals; yeild 73%; M.pt. 320‐323°C FT‐IR (cm^−^^1^): 3217.99 (OH), 1745.25, 1712.67 (C═O, quinazoline‐6,12‐dione), 1609.53, 1504.29 (Ar C═C/C═N), 1309.01 (C‐N), 1207.40, 1097.18 (Ar O‐CH_3_), 1H NMR (500 MHz, DMSO‐*d*6) δ 9.75 (s, 1H), 8.00 (d, *J* = 8.2 Hz, 1H), 7.77 (d, *J* = 2.7 Hz, 1H), 7.61 (d, *J* = 8.5 Hz, 1H), 7.57 (d, *J* = 2.5 Hz, 1H), 7.18 (dd, *J* = 8.5, 2.7 Hz, 1H), 7.00 (dd, *J* = 8.5, 2.6 Hz, 1H), 3.75 (s, 3H), 13C NMR (126 MHz, DMSO) 184.55 (C‐9, lactam C═O), 159.30 (C‐15, Ar‐C‐OH), 155.21 (C‐1, Ar‐C‐OCH_3_), 144.55 (C‐11, quaternary C‐N), 142.67 (C‐4, quaternary C‐N), 139.56 (C‐7, quaternary aromatic C), 139.03 (C‐12, quaternary aromatic C), 131.89 (C‐8, fused quaternary C), 128.10 (C‐2, Ar‐CH), 124.83 (C‐14, Ar‐CH), 124.76 (C‐17, Ar‐CH), 123.50 (C‐3, Ar‐CH), 118.06 (C‐5, Ar‐CH), 113.11 (C‐6, Ar‐CH), 109.55 (C‐16, Ar‐CH), 55.78 (C‐22, O‐CH_3_), HRMS (ESI^+^): *m/z* calculated for C_16_H_10_N_2_O_4_ [M+H]^+^: 295.07, found: 295.0859.

#### 4.1.8 (SJ8)2‐hydroxy‐8‐nitroindolo[2,1‐*b*]quinazoline‐6,12‐dione

It was obtained as solid brown crystals; yeild 68%; M.pt. 315‐320°C; FT‐IR (cm^−^^1^): 3273.20 (OH), 1744.35, 1609.47 (C═O, quinazoline‐6,12‐dione), 1504.91 (NO_2_ asymmetrical), 1309.31 (NO_2_ symmetrical), 1207.50 (C‐N), 764.43 (aromatic C‐H). 1H NMR (500 MHz, DMSO‐*d*6) δ 9.76 (s, 1H), 8.79 (dd, *J* = 8.4, 2.6 Hz, 1H), 8.62 (d, *J* = 2.3 Hz, 1H), 8.16 (d, *J* = 8.7 Hz, 1H), 7.62 (d, *J* = 8.5 Hz, 1H), 7.57 (d, *J* = 3.7 Hz, 1H), 7.16 (dd, *J* = 8.9, 2.5 Hz, 1H), 13C NMR (126 MHz, DMSO) 183.29 (C‐9, lactam C═O), 160.75 (C‐10, lactam C═O), 158.77 (C‐15, Ar–C–OH), 156.69 (C‐11, quaternary C–N), 148.71, 148.05 (C‐1, Ar‐C‐NO_2_), 145.16 (C‐4, quaternary C‐N), 140.85 (C‐7, quaternary aromatic C), 137.71 (C‐12, quaternary aromatic C), 135.47, 133.90, 133.62 (fused quaternary aromatic C), 131.42 (C‐2, Ar‐CH), 129.31 (C‐14, Ar‐CH), 126.42 (C‐17, Ar‐CH), 119.22 (C‐5, Ar‐CH), 114.63 (C‐6, Ar‐CH), HRMS (ESI^+^): *m/z* calculated for C_15_H_9_N_3_O_5_ [M+H]^+^: 309.06, found: 309.0152.

### 4.2 Computational Studies

#### 4.2.1 In Vitro α‐Amylase Inhibition

A colorimetric test based on starch hydrolysis by pig pancreatic α‐amylase was used to assess the synthesized compounds' in vitro α‐amylase inhibitory activity [42]. The experiment was conducted using a 0.02 M phosphate buffer (pH 6.8) containing 0.01 M calcium chloride. The substrate was soluble starch and 0.1% w/v gum acacia solution was used to prepare test compounds and the standard medication Acarbose at concentrations of 10–100 µg/mL. After 10 min of incubation at 37°C, the reaction mixture including starch substrate, test substance and α‐amylase enzyme (2 U/mL) was stopped with 50% v/v acetic acid. A UV–visible spectrophotometer was used to measure the absorbance of the supernatant at 595 nm after the mixtures were centrifuged. The following formula was used to determine the percentage inhibition of α‐amylase activity:

$$\%\;\mathrm{Inhibition}\;=\left(\frac{A_{c}-A_{s}}{A_{c}}\right)\;\times 100$$

where, *A*_C_ indicates the absorbance of the sample or standard whereas *A*_S_ denotes the absorbance of the control. Every experiment was run in triplicate and the mean ± SEM was used to express the results.

#### 4.2.2 Molecular Docking

AutoDockTools 1.5.7 was used for molecular docking studies to assess the selected compounds binding interactions with α‐Amylase (PDB ID: 2QV4) [43]. ChemBioDraw Ultra 12.0 was used to construct the ligand structures, which were then converted into three‐dimensional structures and energy‐minimized using ChemBioDraw 3D Ultra 12.0 [44]. The RCSB Protein Data Bank was used to get the crystal structure of α‐amylase, which was then processed by removing water molecules, adding polar hydrogens and allocating Gasteiger charges [45, 46, 47]. A grid spacing of 0.375 Å was used to generate receptor grids surrounding the active region. The binding orientations, interaction patterns and docking scores of the ligands inside the catalytic pocket were then predicted using docking analysis. For further synthesis and biological testing, compounds with favorable docking scores and noteworthy ligand‐protein interactions were chosen [43].

#### 4.2.3 Molecular Dynamics

GROMACS 2022.3 was used to perform molecular dynamics (MD) simulations of the SJ2‐α‐Amylase complex using the GAFF2 parameters for the ligand and the AMBER99SB‐ILDN force field for the protein. The complex was neutralized using Na^+^ and Cl^−^ counter‐ions after being solvated in a TIP3P explicit water model inside a cubic simulation box while keeping a buffer distance of 10 Å from the protein surface [48]. The steepest descent technique was used to minimize energy for 50 000 steps in order to remove un‐favorable interactions and steric conflicts. Two phases of system equilibration were carried out: NVT ensemble equilibration at 300 K for 100 ps and NPT ensemble equilibration at 1 bar and 300 K for 100 ps. Trajectory coordinates were then captured every 10 ps during a 100 ns production MD simulation with a 2fs integration time step. Using built‐in GROMACS utilities and internal analysis scripts, post‐simulation analyses such as root‐mean‐square deviation (RMSD), root‐mean‐square fluctuation (RMSF), radius of gyration (Rg), solvent‐accessible surface area (SASA), hydrogen bond occupancy, contact map analysis, dynamic cross‐correlation matrix (DCCM) and free energy landscape (FEL) computations were carried out [49].

#### 4.2.4 ADMET Studies

The compounds underwent in silico ADMET profiling to assess their pharmacokinetic behavior, safety, and drug‐likeness characteristics after the ligands were chosen based on their molecular docking scores and binding interactions with α‐Amylase (PDB ID: 2QV4) [50]. ADMET analysis is crucial in ensuring that the chosen compounds have both significant binding affinity and the desirable properties needed for possible antidiabetic medication candidates [51]. With a focus on Lipinski's Rule of Five and topological polar surface area (TPSA) parameters to estimate intestinal absorption and oral bioavailability, the ADMET characteristics were predicted using SwissADME [52]. Compounds that met Lipinski's criteria were deemed to have good oral absorption and appropriate drug‐like behavior. These criteria included molecular weight below 500 Da, log P less than 5, hydrogen bond donors ≤ 5, hydrogen bond acceptors ≤ 10 and TPSA values below 140 Å^2^ [53].

### 4.3 Clinical Relevance and Future Therapeutic Potential of SJ2

SJ2 exhibited comparable α‐amylase inhibitory activity (IC_50_ = 18.49 ± 0.06 µM) with reference drug acarbose (IC_50_ = 16.26 ± 0.02 µM) indicating its potential as a lead structure for further development as antidiabetic drug. However, the present study does not experimentally establish the solubility, oral bioavailability, enzyme selectivity, gastrointestinal tolerability, or in vivo efficacy of SJ2. Therefore, these properties cannot yet be considered therapeutic advantages over acarbose. The in silico ADMET results provide preliminary support for further investigation but should not be interpreted as evidence of clinical pharmacokinetic or safety performance. Future studies should evaluate the aqueous solubility, absorption and pharmacokinetic properties of SJ2, together with selectivity against other digestive and metabolically relevant enzymes and appropriate gastrointestinal tolerability studies. In vivo evaluation of postprandial glucose‐lowering activity and toxicity will also be necessary to determine whether the observed in vitro α‐amylase inhibition can translate into therapeutic benefit. Thus, SJ2 should currently be considered a promising α‐amylase inhibitory lead rather than a clinically validated antidiabetic candidate.

## Author Contributions

**Sahil Jaidka**: conceptualization, data curation, formal analysis, and writing – original draft preparation. **Ankush Kumar**: literature review, data collection, and writing – review and editing. **Thakur Gurjeet Singh**: supervision, validation. **Rohit Bhatia**: project administration, supervision, and final approval of the manuscript.

## Conflicts of Interest

The authors declare no conflicts of interest.

No AI tool was used during preparation of the manuscript.

## Supporting information




**Supporting File 1**: cbdv71694‐sup‐0001‐SuppMat.docx.

## Data Availability

Data sharing not applicable to this article as no datasets were generated or analyzed during the current study.Use of Generative AI and AI‐Assisted Technologies in the Writing Process
