# Supplementary material for: Design, Synthesis, Molecular Dynamics, and In Vitro Evaluation of Isatin‐Based Quinazoline Derivatives as Potential Antidiabetic Agents
Source: Chem Biodivers. 2026 Sep 10;23(9):e71694. doi: 10.1002/cbdv.71694 (PMC13559870; doi:10.1002/cbdv.71694)
Supplement: Supplementary file 1 [file CBDV-23-e71694-s001.docx]

**Supplementary Data:**

**Design, Synthesis, Molecular Dynamics and *in vitro* Evaluation of Isatin Based Quinazoline Derivatives as Potential Antidiabetic Agents**

**Sahil Jaidka¹, Ankush Kumar^1^, Christopher Basumatary^2^, Thakur Gurjeet Singh¹*, Rohit Bhatia¹***

*¹Chitkara College of Pharmacy, Chitkara University, Rajpura, Punjab-140401, India*

*^2^Department of Pharmaceutical Engineering and Technology, IIT BHU, Varanasi, India.*

**Corresponding authors:* [*bhatiarohit5678@gmail.com*](mailto:bhatiarohit5678@gmail.com)*,* [*gurjeet.singh@chitkara.edu.in*](mailto:gurjeet.singh@chitkara.edu.in)

**Table. S1: In Silico ADME and Physicochemical Property Evaluation of Designed Indolo[2,1-*b*]quinazoline Derivatives.**

| **S. No.** | **Carcinogenicity** | **Mutagenicity** | **Physicochemical Properties** | **Water Solubility** | **iLog-P** | **P’cokinetics** | **Synthetic Accessibility** |
| --- | --- | --- | --- | --- | --- | --- | --- |
| **SJ1** | No structural alerts predicted | No structural alerts predicted | MW: 282.68 g/mol; TPSA: 51.96 Å² | Soluble | 2.18 | High GI absorption; BBB permeant | 2.43 |
| **SJ2** | No structural alerts predicted | No structural alerts predicted | MW: 361.58 g/mol; TPSA: 51.96 Å² | Moderately soluble | 2.48 | High GI absorption; BBB permeant | 2.50 |
| **SJ3** | No structural alerts predicted | No structural alerts predicted | MW: 312.71 g/mol; TPSA: 61.19 Å² | Soluble | 2.45 | High GI absorption; BBB permeant | 2.54 |
| **SJ4** | No structural alerts predicted | No structural alerts predicted | MW: 327.68 g/mol; TPSA: 97.78 Å² | Soluble | 1.68 | High GI absorption; Not BBB permeant | 2.63 |
| **SJ5** | No structural alerts predicted | No structural alerts predicted | MW: 264.24 g/mol; TPSA: 72.19 Å² | Soluble | 1.55 | High GI absorption; BBB permeant | 2.41 |
| **SJ6** | No structural alerts predicted | No structural alerts predicted | MW: 343.13 g/mol; TPSA: 72.19 Å² | Moderately soluble | 1.86 | High GI absorption; BBB permeant | 2.49 |
| **SJ7** | No structural alerts predicted | No structural alerts predicted | MW: 294.26 g/mol; TPSA: 81.42 Å² | Soluble | 1.81 | High GI absorption; Not BBB permeant | 2.58 |
| **SJ8** | No structural alerts predicted | No structural alerts predicted | MW: 309.23 g/mol; TPSA: 118.01 Å² | Soluble | 1.08 | High GI absorption; Not BBB permeant | 2.61 |

- **Toxicity Profiling:**

**Table. S2: In Silico Toxicity Prediction Profile of Designed Indolo[2,1-*b*]quinazoline Derivatives.**

| **Compound ID** | **AMES Toxicity** | **Max. Tolerated Dose (Human) *(log mg/kg/day)*** | **hERG I Inhibitor** | **hERG II Inhibitor** | **Oral Rat Acute Toxicity (LD₅₀) *(mol/kg)*** | **Oral Rat Chronic Toxicity (LOAEL) *(log mg/kg bw/day)*** | **Hepatotoxicity** |
| --- | --- | --- | --- | --- | --- | --- | --- |
| **SJ1** | **Yes** | -0.183 | No | No | 2.339 | 1.394 | No |
| **SJ2** | No | -0.596 | No | No | 2.307 | 1.414 | No |
| **SJ3** | No | -0.205 | No | No | 2.479 | 1.418 | No |
| **SJ4** | **Yes** | -0.723 | No | No | 2.769 | 1.120 | **Yes** |
| **SJ5** | **Yes** | 0.021 | No | **Yes** | 1.969 | 1.359 | No |
| **SJ6** | No | -0.013 | No | **Yes** | 2.182 | 1.296 | No |
| **SJ7** | No | 0.158 | No | **Yes** | 2.442 | 1.140 | No |
| **SJ8** | **Yes** | -0.200 | No | No | 2.379 | 1.259 | No |

- **Synthetic Outcomes:**

**Compound SJ1:**

**Fig. S1.1: IR of compound SJ1**

**Fig. S1.2: ^1^H-NMR of compound SJ1**

**Fig. S1.3: ^13^C-NMR of compound SJ1**

**Fig. S1.4: ^1^H-NMR of compound SJ1**

- **Compound SJ2:**

**Fig. S2.1: IR of compound SJ2**

**Fig. S2.2: ^1^H-NMR of compound SJ2**

**Fig. S2.2: ^13^C-NMR of compound SJ2**

**Fig. S2.4: HRMS of compound SJ2**

- **Compound SJ3:**

**Fig. S3.1: IR of compound SJ3**

**Fig. S3.2: ^1^H-NMR of compound SJ3**

**Fig. S3.3: ^13^C-NMR of compound SJ3**

**Fig. S3.4: HRMS of compound SJ3**

- **Compound SJ4:**

**Fig. S4.1: IR of compound SJ4**

**Fig. S4.2: ^1^H-NMR of compound SJ4**

**Fig. S4.3: ^13^C-NMR of compound SJ4**

**Fig. S4.4: HRMS of compound SJ4**

- **Compound SJ5**

**Fig. S5.1: IR of compound SJ5**

**Fig. S5.2: ^1^H-NMR of compound SJ5**

**Fig. S5.3: ^13^C-NMR of compound SJ5**

**Fig. S5.4: HRMS of compound SJ5**

- **Compound SJ6:**

**Fig. S6.1: IR of compound SJ6**

**Fig. S6.2: ^1^H-NMR of compound SJ6**

**Fig. S6.3: ^13^C-NMR of compound SJ6**

**Fig. S6.4: HRMS of compound SJ6**

- **Compound SJ7**

**Fig. S7.1: IR of compound SJ7**

**Fig. S7.2: ^1^H-NMR of compound SJ7**

**Fig. S7.3: ^13^C-NMR of compound SJ7**

**Fig. S7.4: HRMS of compound SJ7**

- **Compound SJ8:**

**Fig. S8.1: IR of compound SJ8**

**Fig. S8.2: ^1^H-NMR of compound SJ8**

**Fig. S8.3: ^13^C-NMR of compound SJ8**

**Fig. S8.4: HRMS of compound SJ8**

- **Molecular Dynamics:**

**Fig. S9:** Overlaid RMSD of protein backbone (blue) and SJ2 ligand (red) during 100 ns MD simulation. The stable protein RMSD (0.10-0.18 nm) contrasted with ligand RMSD (0.2-0.8 nm) demonstrates adaptive ligand sampling within a rigid active site without dissociation.

**Fig. S10:** Solvent accessible surface area (SASA) of α-amylase during 100 ns MD simulation with SJ2. Stable SASA (198-202 nm²) confirms no major changes in protein surface exposure, supporting intact complex formation.

**Fig. S11:** Secondary structure evolution of α-amylase over 100 ns MD simulation with SJ2 (DSSP analysis). Persistent horizontal bands confirm that α-helices (red) and β-strands (orange) are maintained throughout the simulation, confirming SJ2 does not induce secondary structure disruption

**Fig. S12:** Protein-ligand hydrogen bond analysis during 100 ns MD simulation.), H-bond frequency histogram showing 1–2 H-bonds as the dominant states (>70% of simulation frames).

**Fig. S13:** Protein-ligand atomic contacts (<0.35 nm cutoff) during 100 ns MD simulation. The persistent contact count (~40-60) confirms SJ2 remains fully engaged within the α-amylase active site throughout the simulation.
